# Thermodynamic flow of radiative induced magneto modified Maxwell Sutterby fluid model at stretching sheet/cylinder

**DOI:** 10.1038/s41598-023-40843-w

**Published:** 2023-09-25

**Authors:** Nadeem Abbas, Wasfi Shatanawi, Fady Hasan, Zead Mustafa

**Affiliations:** 1 Department of Mathematics and Sciences, College of Humanities and Sciences, Prince Sultan University, 11586 Riyadh, Saudi Arabia; 2 Department of Medical Research, China Medical University Hospital, China Medical University, Taichung, 40402 Taiwan; 3 Department of Mathematics, Faculty of Science, The Hashemite University, P.O Box 330127, Zarqa, 13133 Jordan

**Keywords:** Mathematics and computing, Nanoscience and technology

## Abstract

A steady flow of Maxwell Sutterby fluid is considered over a stretchable cylinder. The magnetic Reynolds number is considered very high and induced magnetic and electric fields are applied on the fluid flow. Joule heating and radiation impacts are studied under the temperature-dependent properties of the liquid. Having the above assumptions, the mathematical model has been evolving via differential equations. The differential equations are renovated in the dimensionless form of ordinary differential equations using the appropriate transformations. The numerical results have been developed employing numerical techniques on the ordinary differential equations. The impact of involving physical factors on velocity, induced magneto hydrodynamic, and temperature function is debated in graphical and tabular form. The velocity profile is boosted by thicker momentum boundary layers, which are caused by higher values of the magnetic field factor. So, the fluid flow becomes higher velocity due to enlarging values of the magnetic field factor. Heat transfer factor and friction at surface factor boosted up for increment of ${\gamma }_{0}$ (Magnetic field factor). The ${\gamma }_{0}$(Magnetic field factor) is larger which better-quality of heat transfer at surface and also offered the results of friction factor boosting up in both cases of stretching sheet/cylinder. The ${\lambda }_{0}$(Magnetic Prandtl number) increased which provided better-quality of heat transfer at surface.

## Introduction

Flow over stretching cylinder has a large number of applications in the engineering and industrial fields including the production and extraction of glass fiber manufacturing, thermoplastic melt-spinning rubber sheet, and so on. Scholars are very interested in the perception of fluid flow throughout the cylinder. Takahashi et al.^1^ questioned the stretchable cylinder by the MHD thermodynamics of temporal variation and dynamo action. Amkadni and Azzouzi^2^ discussed the moving stretchable cylinder by implementing the magnetic hydrodynamic effect. Ishak et al.^3^ highlighted the impression of hydrodynamic flow at the stretchable cylinder. The numerical consequences have been settled under the dimensionless system of differential equations. Mukhopadhyay^4^ studied the stretchable cylindrical surface implementing the slip effects of magnetic hydrodynamic boundary layer flow. The viscous liquid is considered to discuss the impact of partial slip at the surface of a cylinder. Tamoor et al.^5^ deliberated the stretchable cylinder for the Casson MHD fluid flow. The joule heating and viscous dissipation are considered to reveal the outcomes. Sohail and Naz^6^ studied the modified model of the non-viscous liquid model using the magnetic hydrodynamic at a stretchable cylinder. The Sutterby model has been argued to analyze the flow impacts. Abbas et al.^7^ discussed the inclined magnetic hydrodynamic flow of hybrid nanomaterial liquid at a stretchable cylinder. Mandal et al.^8^ discussed the impact of inclined radiation flow for nanomaterial liquid using microorganisms with the stratification of thermo-solutal. Researchers have debated about the stretchable cylinder using the various assumptions for various fluid models see Refs.^9, 10^.

Numerous studies on non-Newtonian hydrodynamics, including magnetohydrodynamics, have been reported in the literature. Takashima^11^ deliberated the Maxwell liquid model using the magnetic field and thermal instability impacts. Sengupta and Bhattacharyya^12^ highlighted the impression of the viscoelastic flow of Maxwell liquid in the regular channel in the presence of a magnetic field impression. The pressure gradient of transient and periodic impact has been considered. Renardy and Renardy^13^ discussed the upper convected flow of Maxwell liquid using the Couette flow. The linear stability results are presented. Fetecau et al.^14^ emphasized the impact of the Maxwell model using the time-dependent flow at a stretching sheet. The fractional derivative has been implemented to achieve results. Abel et al.^15^ considered the Maxwell fluid model using the upper convected at a stretchable sheet. The magnetic hydrodynamic flow has been studied in the present analysis. Hsiao^16^ debated the composite study of the electrical magnetic hydrodynamic of the Maxwell liquid model at a stretchable surface. Radiative viscous dissipation has been studied. Nadeem et al.^17^ debated the flow of the stagnation region of Maxwell micropolar liquid at the Riga sheet. Nadeem et al.^18^ deliberated the heat and mass transfer of Maxwell micropolar liquid having the stagnation point at a Riga sheet. Authors (Refs.^19, 20^) have settled ideas about Maxwell liquid via stretchable surfaces.

The Sutterby liquid was discussed by Batra and Eissa^21^ in early time. Helical flow is considered to analyze the various effects of involving physical. Manglik and Fang^22^ debated the impression of non-Newtonian liquid using the power law rheology with the thermal condition at the boundary. Jain et al.^23^ debated the Sutterby liquid model using natural convection. The isothermal surface has been considered in the presence of steady flow. Eldesoky et al.^24^ debated the influence of particulate suspension in between channels. Ahmad et al.^25^ chemical reactive flow of Sutterby liquid model of squeezing having thermal radiation. The maxed convection under double stratification at a stretchable surface has been studied. Mir et al.^26^ debated the impact of the Sutterby liquid model by implementing the theory of Cattaneo–Christov over the stretchable sheet. The thermal stratified for heat absorption/ generation was considered in their analysis. Sabir et al.^27^ debated the impression of Sutterby liquid for stagnation region numerically. Sutterby fluid model have been debated in various assumptions at stretching surafce (see Refs.^28–31^).

Classical dynamic is subclass of magnetohydrodynamics (MHD) which studied about eletrical conducting fluid in the occurrence of magnetic field. Dormy et al.^32^ disputed the impression of magnetic hydrodynamic at a spherical sheet. Beg et al.^33^ discussed the electrically conducting induced magnetohydrodynamic having laminar flow. Ajao et al.^34^ debated the induced magnetohydrodynamic with an electric field and cylindrical magnet. Gireesha et al.^35^ discussed the nanomaterial flow of induced magnetohydrodynamic at a stretchable sheet. The melting effects of Brownian motion and thermophoresis are debated in their analysis. Hanaya et al.^36^ argued the influence of the hybrid nanomaterial flow of micropolar liquid at a curved sheet in the presence of induced magnetohydrodynamics. Khan et al.^37^ debated the influence of induced magneto-hydrodynamic flow chemically reactive nanomaterial liquid at a nonlinear stretchable sheet. Nawaz et al.^38^ emphasized the effects of MHD with electrical casson nanomaterial fluid flow. Shatnawi et al.^39^ debated the inspiration of sutterby-induced MHD flow at stretchable cylinder. In the present days, few authors settled ideas using the induced magnetohydrodynamic flow for several fluid models under the flow factors (see Refs.^40–42^).

From the above literature, the Maxwell sutteby fluid model is considered at the stretchable cylinder. Viscous dissipation and Darcy resistance influences have been deliberated. The induced magnetic field is applied to the flow and considered a high Reynolds number. The variable thermal conductivity and radiative impacts are studied. The system of PDE’s has been developed after applying the boundary layer approximation on the governing equations. The governing equation has been developed under the flow region. The PDE’s become dimensionless (ODE’s) using suitable transformations. The ODE’s are solved through a numerical approach. The governing physical factors have been revealed through tabular and graphical forms. These results are unique and no one discussed them before it.

## Mathematical formulation

**Figure 1:**
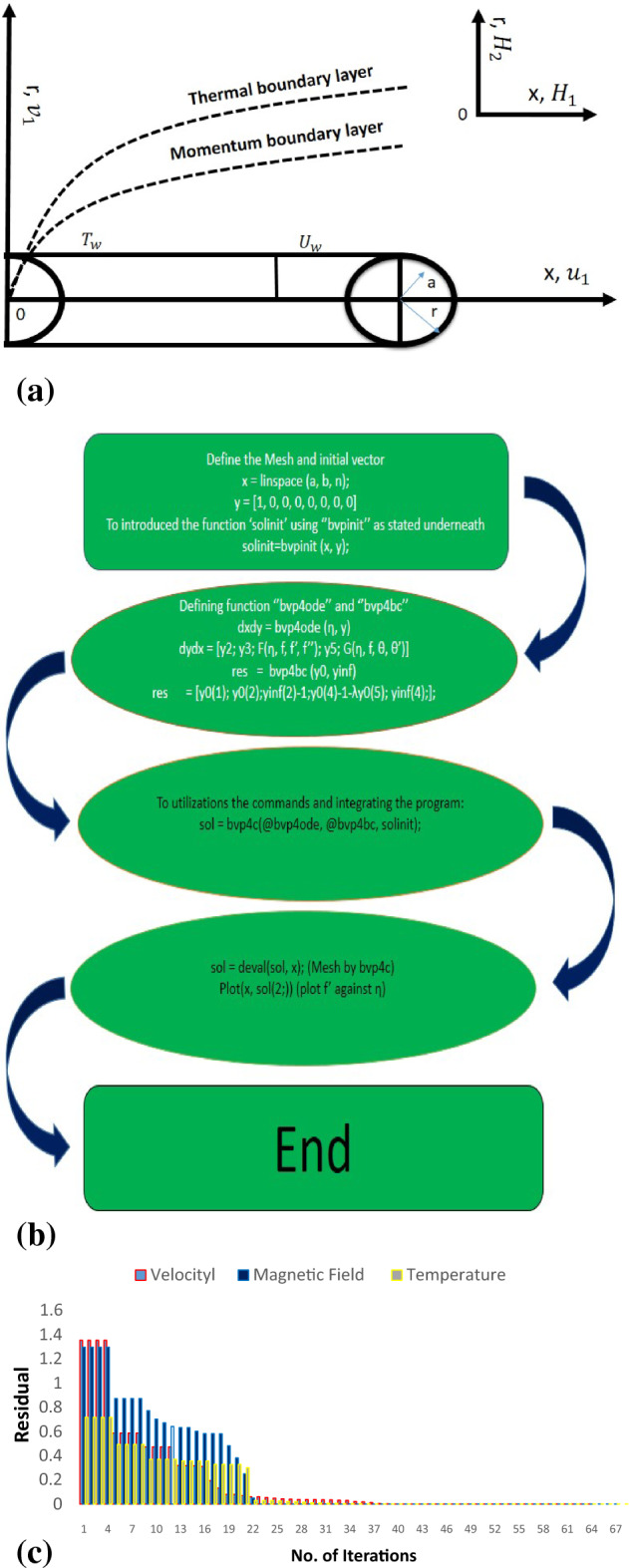
(**a**) Flow pattern of Maxwell Sutterby fluid at stretching cylinder/sheet. (**b**) Description of the numerical scheme. (**c**) Description of the numerical scheme.

Steady flow of incompressible is considered at a stretchable cylinder/sheet. The flow of Maxwell Sutterby fluid pattern is offered in Fig. 1a. Variable thermal conductivity is publicised as $K\left(T\right)={k}_{\infty }(1+\epsilon\theta \left(\zeta \right))$. The induced magnetic field is deliberated with Sutterby fluid in the presence of radiations. The mathematical model have been established under the boundary layer estimates. The flow assumptions are:

- Maxwell fluid
- Sutterby fluid
- Induced magnetic field
- Stretching cylinder/sheet
- Radiation and variable thermal conductivity
- Joule heating

The developing model as following (see Refs.^6, 43, 44^):

1 $$\frac{\partial {v}_{1}}{\partial r}+\frac{{v}_{1}}{r}+\frac{\partial {u}_{1}}{\partial x}=0,$$

2 $$\frac{\partial {H}_{2}}{\partial r}+\frac{{H}_{2}}{r}+\frac{\partial {H}_{1}}{\partial x}=0,$$

3 $${u}_{1}\frac{\partial {u}_{1}}{\partial x}+{v}_{1}\frac{\partial {u}_{1}}{\partial r} -\frac{{\mu }_{e}}{4\pi \rho }\left({H}_{1}\frac{\partial {H}_{1}}{\partial x}+{H}_{2}\frac{\partial {H}_{1}}{\partial r}\right)+\tau \left({{u}_{1}}^{2}\frac{{\partial }^{2}{u}_{1}}{\partial {x}^{2}}+{{v}_{1}}^{2}\frac{{\partial }^{2}{u}_{1}}{\partial {r}^{2}}+2{v}_{1}{u}_{1}\frac{{\partial }^{2}{u}_{1}}{\partial r\partial x}\right)=\left(\frac{{\upsilon }_{f}}{2}\right)\frac{{\partial }^{2}{u}_{1}}{\partial {r}^{2}}+\left(\frac{{\upsilon }_{f}}{2}\right)\frac{1}{r}\frac{\partial {u}_{1}}{\partial r}-\left(\frac{{\upsilon }_{f}m{{a}_{0}}^{2}}{4}\right){\left(\frac{\partial {u}_{1}}{\partial r}\right)}^{2}\frac{{\partial }^{2}{u}_{1}}{\partial {r}^{2}}+\frac{1}{\rho_f}R_z,$$

4 $${u}_{1}\frac{\partial {H}_{1}}{\partial x}+ {v}_{1}\frac{\partial {H}_{1}}{\partial r}- {H}_{1}\frac{\partial u}{\partial x}- {H}_{2}\frac{\partial u}{\partial r}= {\eta }_{0}\left(\frac{{\partial }^{2}{H}_{1}}{\partial {r}^{2}}+\frac{1}{r}\frac{\partial {H}_{1}}{\partial r}\right),$$

5 $${u}_{1}\frac{\partial T}{\partial x}+ {v}_{1}\frac{\partial T}{\partial r}=\frac{1}{ \rho {c}_{p}}\frac{1}{r}\frac{\partial }{\partial r}\left(K\left(T\right)r\frac{\partial T}{\partial r}\right)+\frac{1}{ \rho {c}_{p}}{Q}_{0}\left(T- {T}_{\infty }\right)-\frac{1}{ \rho {c}_{p}}\frac{1}{r}\frac{\partial ({q}_{r}r)}{\partial r}+\frac{\mu }{ \rho {c}_{p}}{\left(\frac{\partial {u}_{1}}{\partial r}\right)}^{2}.$$

With the following suitable boundary conditions:

6 $$\begin{gathered} u_{1} = U_{w} , v_{1} = 0, H_{1} = 0, H_{2} = 0, T - T_{w} = 0, at\, r \to a, \hfill \\ u_{1} \to 0, H_{1} \to H_{e} , T \to { }T_{\infty } , as\, r \to \infty . \hfill \\ \end{gathered}$$

Transformations are introduced (see Refs.^6, 43, 44^):

7 $${u}_{1}= \frac{1}{r}\frac{\partial \psi }{\partial r}, {v}_{1}= -\frac{1}{r}\frac{\partial \psi }{\partial x}, {H}_{1}= \frac{1}{r}\frac{\partial {\psi }_{1}}{\partial r} , {H}_{2}=-\frac{1}{r} \frac{\partial {\psi }_{1}}{\partial x}, \eta =\frac{{r}^{2}-{a}^{2}}{2a}\sqrt{\frac{{U}_{w}}{{\upsilon }_{f}x}}, T= {T}_{\infty }+\left({T}_{w}- {T}_{\infty }\right)\uptheta \left(\eta \right).$$

${\psi }_{1}$ $-$ is the magnetic stream function and $\psi$ velocity stream function. The dimensionless form become as

8 $$\left(1+2\eta \varpi \right){F}^{{\prime}{\prime}{\prime}}\left(\eta \right)+2{F}^{{\prime}{\prime}}\left(\eta \right)F\left(\eta \right)-{\left({F}{\prime}\left(\eta \right)\right)}^{2}-\frac{{\alpha }_{0}}{2}{\left(1+2\eta \varpi \right)}({{F}^{{\prime}{\prime}}\left(\eta \right))}^{2} ({\left(1+2\eta \varpi \right)}{F}^{{\prime}{\prime}{\prime}}\left(\eta \right)+\varpi{F}^{{\prime}{\prime}})+\frac{1}{12}{\alpha }_{1}{\left({F}^{{\prime}{\prime}}\left(\eta \right)\right)}^{2}{F}{\prime}\left(\eta \right)+{\gamma }_{0}\left({G}{\prime}\left(\eta \right){G}{\prime}\left(\eta \right)-G\left(\eta \right){G}^{{\prime}{\prime}}\left(\eta \right)\right)+{\beta }_{1}\left(2F\left(\eta \right){F}{\prime}\left(\eta \right){F}^{{\prime}{\prime}}\left(\eta \right)-F\left(\eta \right)F\left(\eta \right){F}^{{\prime}{\prime}{\prime}}\left(\eta \right)-\frac{\varpi }{\left(1+2\eta \varpi \right)}F\left(\eta \right)F\left(\eta \right){F}^{{\prime}{\prime}}\left(\eta \right)\right)=0,$$

9 $${\lambda }_{0}\left(1+2\eta \varpi \right){G}^{{\prime}{\prime}{\prime}}\left(\eta \right)+{\lambda }_{0}\varpi {G}^{{\prime}{\prime}}\left(\eta \right)+G\left(\eta \right){F}^{{\prime}{\prime}}\left(\eta \right)-{G}{\prime}{\prime}\left(\eta \right)F\left(\eta \right)=0,$$

10 $$\left(1+\epsilon \theta \left(\eta \right)+\frac{4}{3}Rd\right)\left(1+2\eta \varpi \right){\theta }^{{\prime}{\prime}}(\eta )+\left(PrF\left(\eta \right)+\varpi +\epsilon \varpi \theta \left(\eta \right)+\frac{4}{3}Rd\varpi \right){\theta }{\prime}\left(\eta \right)+Pr{Q}_{1}\theta \left(\eta \right)+PrEc{\left({F}^{{\prime}{\prime}}\left(\eta \right)\right)}^{2}=0.$$

With boundary condition

11 $$\begin{gathered} F\left( 0 \right) = 0,F^{\prime } \left( 0 \right) = 1,F^{\prime } \left( \infty \right) = 1,G\left( 0 \right) = 0,G^{\prime } \left( 0 \right) = 0,G^{\prime } \left( \infty \right) = 1, \hfill \\ \theta \left( 0 \right) - 1 = 0,\,\theta \left( \infty \right) = 0. \hfill \\ \end{gathered}$$

where, curvature ($\varpi$), ${\beta }_{1}$(Maxwell fluid factor), ${\gamma }_{0}$(Magnetic field factor), ${\alpha }_{0}$(Sutterby fluid factor), ${\alpha }_{1}$(Darcy resistant), $\epsilon$(variable thermal conductivity), ${\lambda }_{0}$(Magnetic Prandtl number), $Ec$(Eckert number), $Pr$(Prandtl number), ${Q}_{1}$(Heat generation) and $Rd$(radiation factor).

Where, ${\beta }_{1}=\frac{{U}_{w}\tau }{l}$ (Maxwell fluid factor), $\varpi =\frac{1}{a}\sqrt{\frac{{\upsilon }_{f}l}{{U}_{w}}}$ (Curvature parameter)$,$ ${\gamma }_{0}$ (Magnetic field factor), ${\alpha }_{1}$ (Darcy resistant factor), ${\lambda }_{0}=\frac{{\eta }_{0}}{{\upsilon }_{f}}$ (Magnetic Prandtl number), $Pr=\frac{{\upsilon }_{f}}{{\alpha }_{f}}$ (Prandtl number), $Ec=\frac{{{U}_{w}}^{3}}{{C}_{p}({T}_{w}-{T}_{\infty })}$ (Eckert number), $Rd=\frac{16{\sigma }^{*}{{\check{T}}_{\infty }}^{3}}{3{k}_{\infty }{k}^{*}}$ (Radiation factor) and ${Q}_{1}$ (Heat generation). The physical factors of the flow assumptions are presented as:

12 $${N}_{u}=\frac{x{\left(1+\frac{4}{3}Rd\right)\left[\frac{\partial T}{\partial r}\right]}_{r=a}}{k\left(T-{T}_{w}\right)},$$

The dimensionless form is presented as

13 $${{N}^{n}}_{u}=-{\left(1+\frac{4}{3}Rd\right)\frac{\partial \theta }{\partial \eta }}_{\eta \to 0}.$$

## Numerical procedure

The above differential system solved through numerical. The 4^th^ order R-K-F- technique, which is the numerical scheme using MATLAB software programs. The convergence conditions were unquestionably accepted as a value of ${10}^{-6}$. The primary choice of suitable finite values of ${\eta }_{\infty }$. Boundary layer analysis takes the typical finite value of ${\eta }_{\infty }$ as ${\eta }_{7}$, which satisfies our problem assumptions. Based on the values of ${\eta }_{\infty }=7$, our results are consistent with the adjusted asymptomatic values of the numerical solution. The following procedure is as following:

14 $$\begin{gathered} F\left( \eta \right) = Y\left( 1 \right);F^{\prime } \left( \eta \right) = Y\left( 2 \right);F^{{\prime \prime }} \left( \eta \right) = Y\left( 3 \right);F^{{\prime \prime \prime }} \left( \eta \right) = YY1; \hfill \\ G\left( \eta \right) = Y\left( 4 \right);G^{\prime } \left( \eta \right) = Y\left( 5 \right);G^{{\prime \prime }} \left( \eta \right) = Y\left( 6 \right);G^{{\prime \prime \prime }} \left( \eta \right) = YY2; \hfill \\ \theta \left( \eta \right) = Y\left( 7 \right);\theta ^{\prime } \left( \eta \right) = Y\left( 8 \right);\theta ^{{\prime \prime }} \left( \eta \right) = YY3; \hfill \\ \end{gathered}$$

15 $$YY1=\frac{-1}{\left(1+2{\eta}\varpi \right)-\frac{{\alpha }_{0}}{2}{\left(1+2{\eta}\varpi \right)}^{2} Y\left(3\right)Y\left(3\right)-{\beta }_{1}Y\left(1\right)Y\left(1\right)}\left(2Y\left(1\right)Y\left(3\right)-\frac{{\alpha }_{0}}{2}{\varpi}{\left(1+2{\eta}\varpi \right)} Y\left(3\right)Y\left(3\right)Y\left(3\right)-Y\left(2\right)Y\left(2\right)+\frac{1}{12}{\alpha }_{1}Y\left(3\right)Y\left(2\right)Y\left(3\right)+{\gamma }_{0}\left(Y\left(5\right)Y\left(5\right)-Y\left(4\right)Y\left(6\right)\right)+{\beta }_{1}\left(2Y\left(1\right)Y\left(2\right)Y\left(3\right)-\frac{\varpi }{\left(1+2{\eta}\varpi \right)}Y\left(1\right)Y\left(1\right)Y\left(3\right)\right)\right);$$

16 $$YY2=\frac{-1}{{\lambda }_{0}\left(1+2{\eta}\varpi \right)}\left(2{\lambda }_{0}\varpi Y\left(6\right)+Y\left(4\right)Y\left(3\right)-Y\left(4\right)Y\left(6\right)\right);$$

17 $$YY3=\frac{Pr}{\left(1+\epsilon Y\left(7\right)+\frac{4}{3}Rd\right)\left(1+2{\eta}\varpi \right)}\left(\left(PrY\left(1\right)+\varpi +\epsilon \varpi Y\left(7\right)+\frac{4}{3}Rd\varpi \right)Y\left(8\right)+Pr{Q}_{1}Y\left(7\right)+PrEcY\left(3\right)Y\left(3\right)\right)$$

With boundary condition

18 $$Y0\left(1\right);Y0\left(2\right)-1;Yinf\left(2\right);Y0\left(4\right);Y0\left(5\right);Yinf\left(5\right)-1; Y0\left(7\right);-1; Yinf\left(7\right);$$

System of ODE’s is explained implementing the 4^th^ order R-K-F-scheme. The tolerance error ${10}^{-6}$ is greater than boundary residuals, and the system of differential equations is convergence. The method is repeated unless which achieved the requirement of convergence basis. The residual boundaries are presented as:

$${\widetilde{Y}}_{1}= \left|{Y}_{2}\left(\infty \right)-\widehat{{Y}_{2}}\left(\infty \right)\right|,$$

$${\widetilde{Y}}_{2}= \left|{Y}_{5}\left(\infty \right)-\widehat{{Y}_{5}}\left(\infty \right)\right|.$$

$${\widetilde{Y}}_{3}= \left|{Y}_{7}\left(\infty \right)-\widehat{{Y}_{7}}\left(\infty \right)\right|.$$

Here, $\widehat{{Y}_{2}}\left(\infty \right)$, $\widehat{{Y}_{5}}\left(\infty \right)$ and $\widehat{{Y}_{7}}\left(\infty \right)$ are calculated boundary values. The description of numerical scheme is presented in Fig. 1b. The residual error is calculated in Fig. 1c.

## Results and discussion

**Figure 2:**
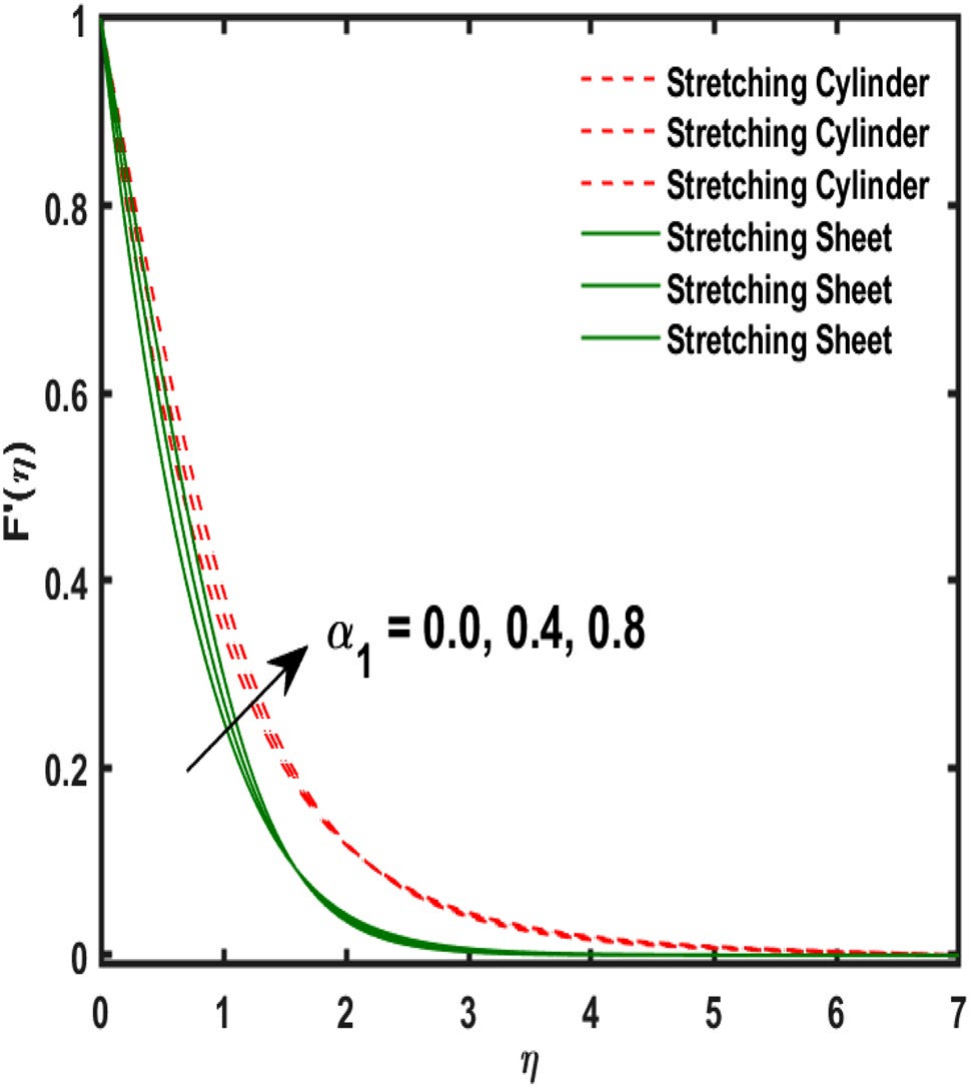
Variation of ${\alpha }_{1}$ and $F{\prime}(\eta )$.

**Figure 3:**
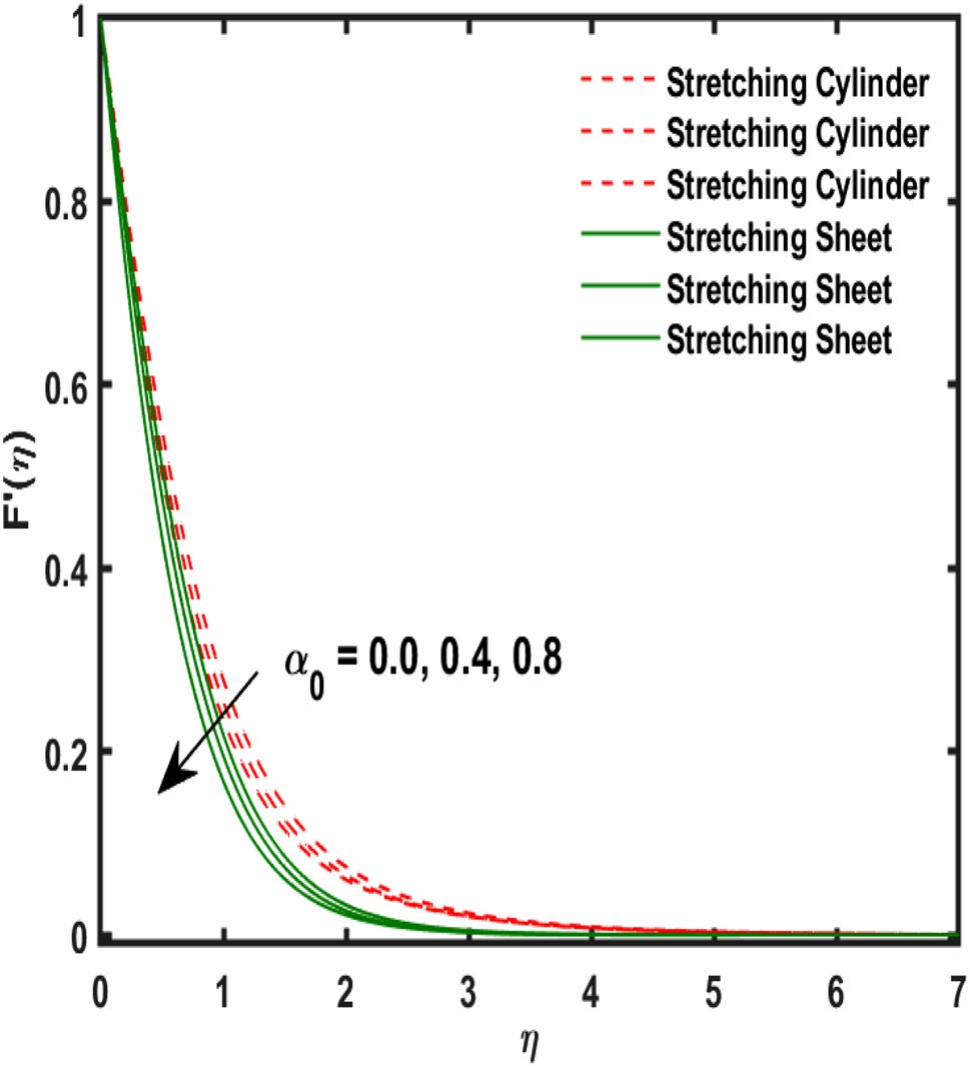
Variation of ${\alpha }_{0}$ and $F{\prime}(\eta )$.

**Figure 4:**
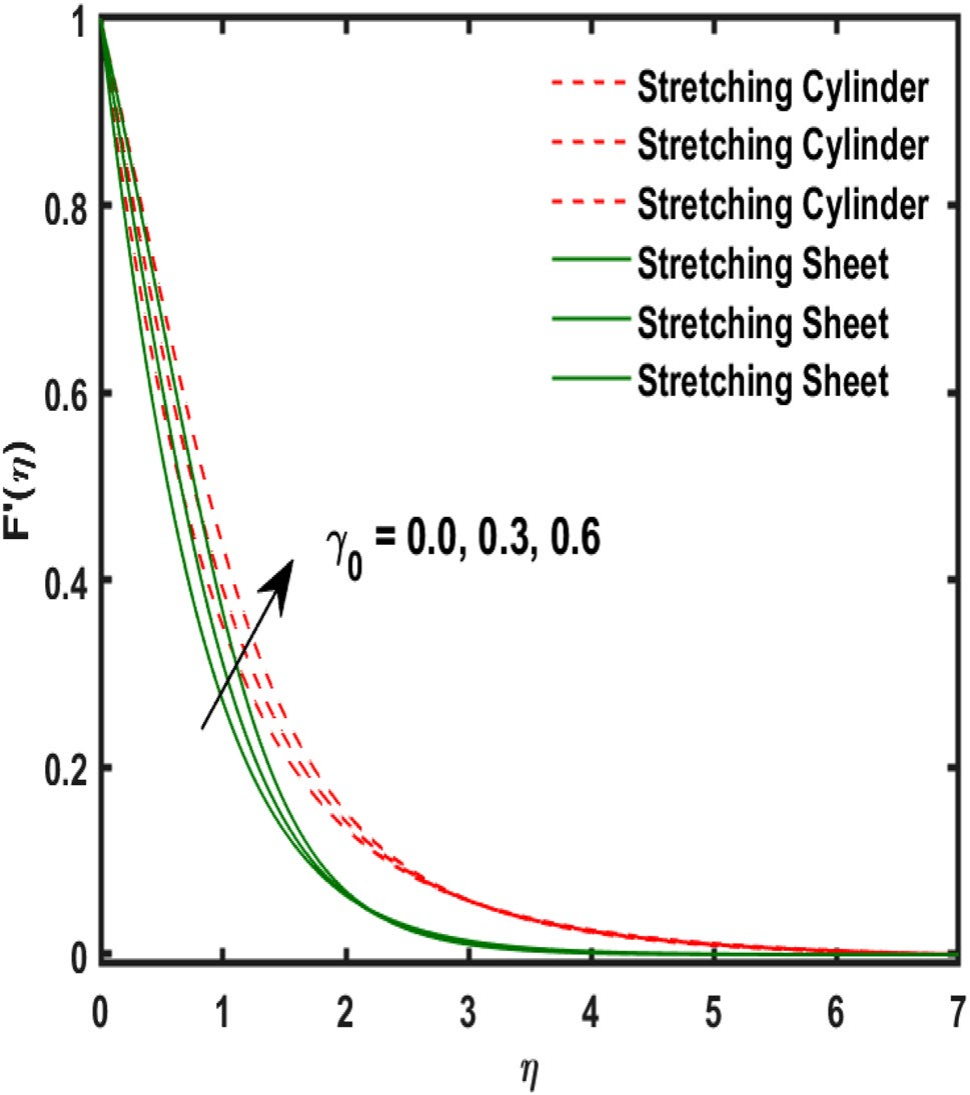
Variation of ${\gamma }_{0}$ and $F{\prime}(\eta )$.

**Figure 5:**
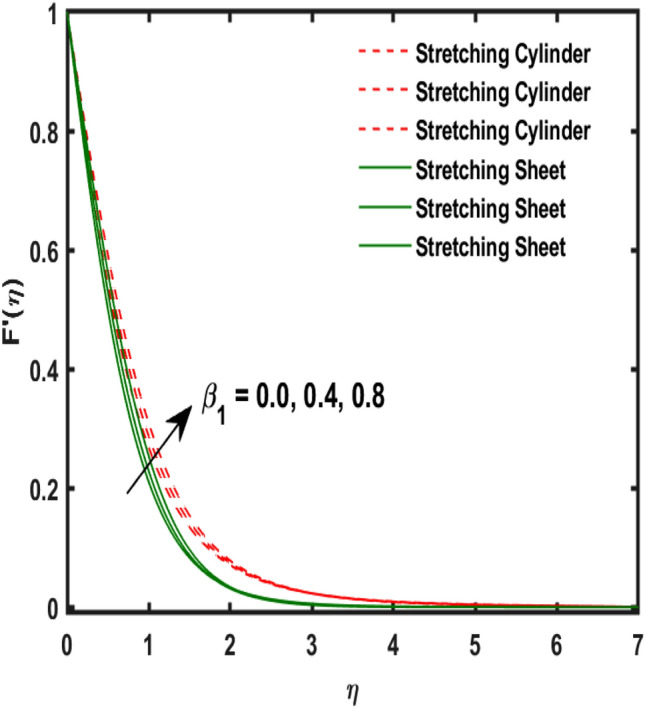
Variation of ${\beta }_{1}$ and $F{\prime}(\eta )$.

**Figure 6:**
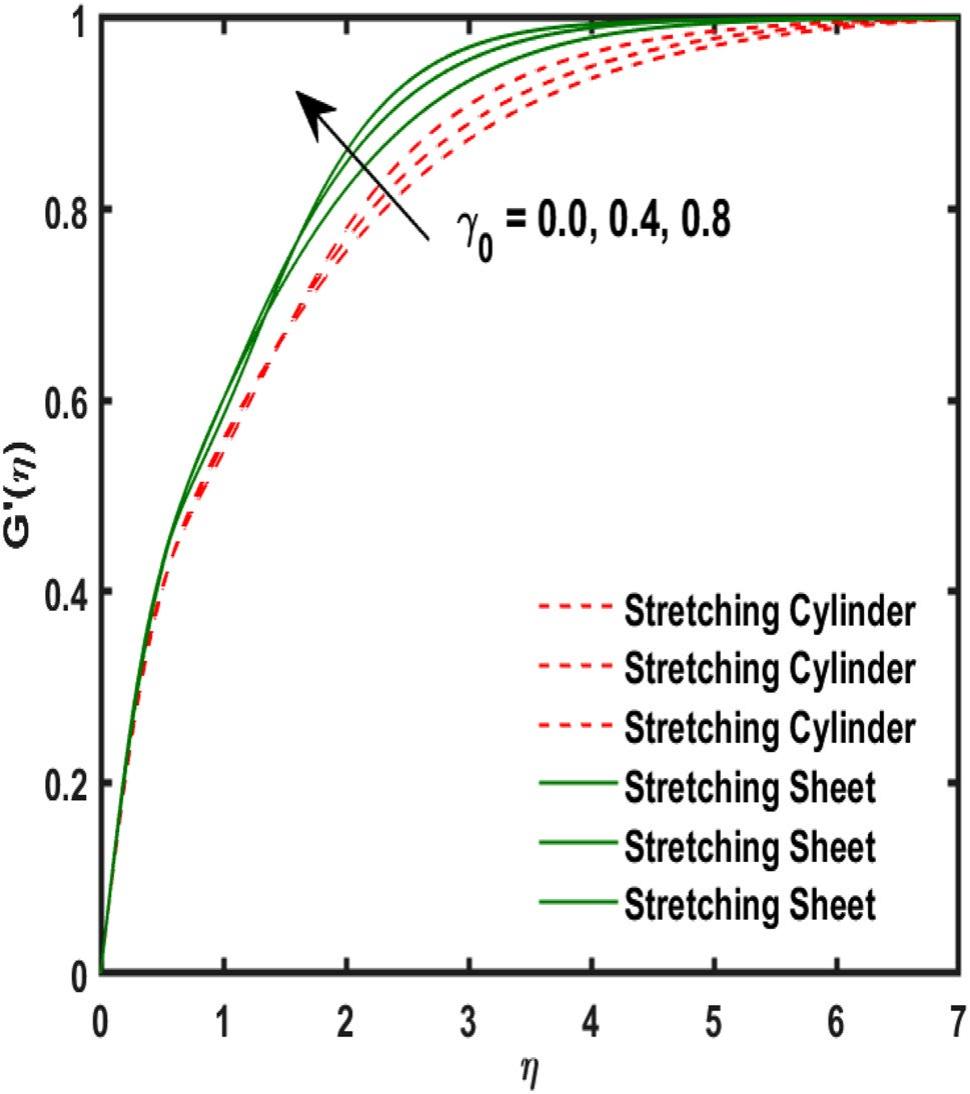
Variation of ${\gamma }_{0}$ and $G{\prime}(\eta )$.

**Figure 7:**
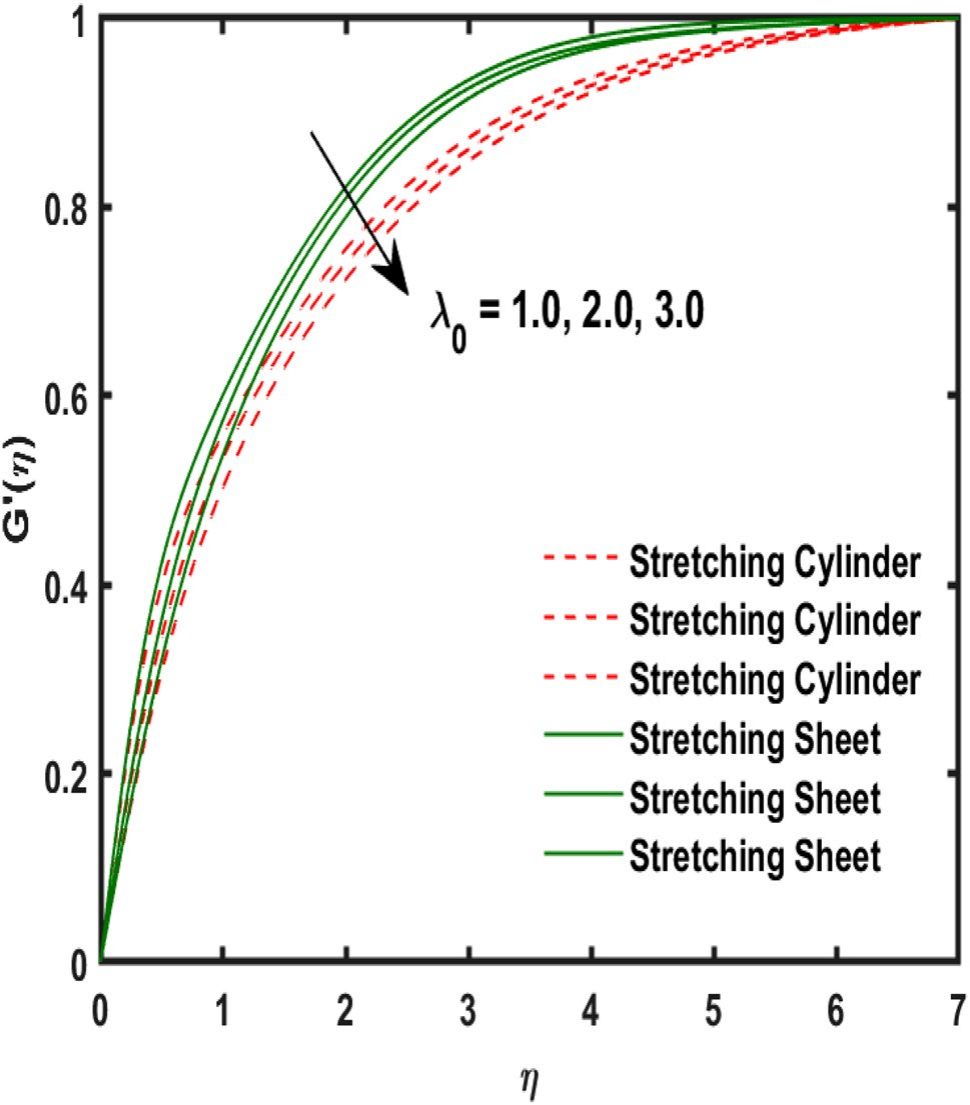
Variation of ${\lambda }_{0}$ and $G{\prime}(\eta )$.

**Figure 8:**
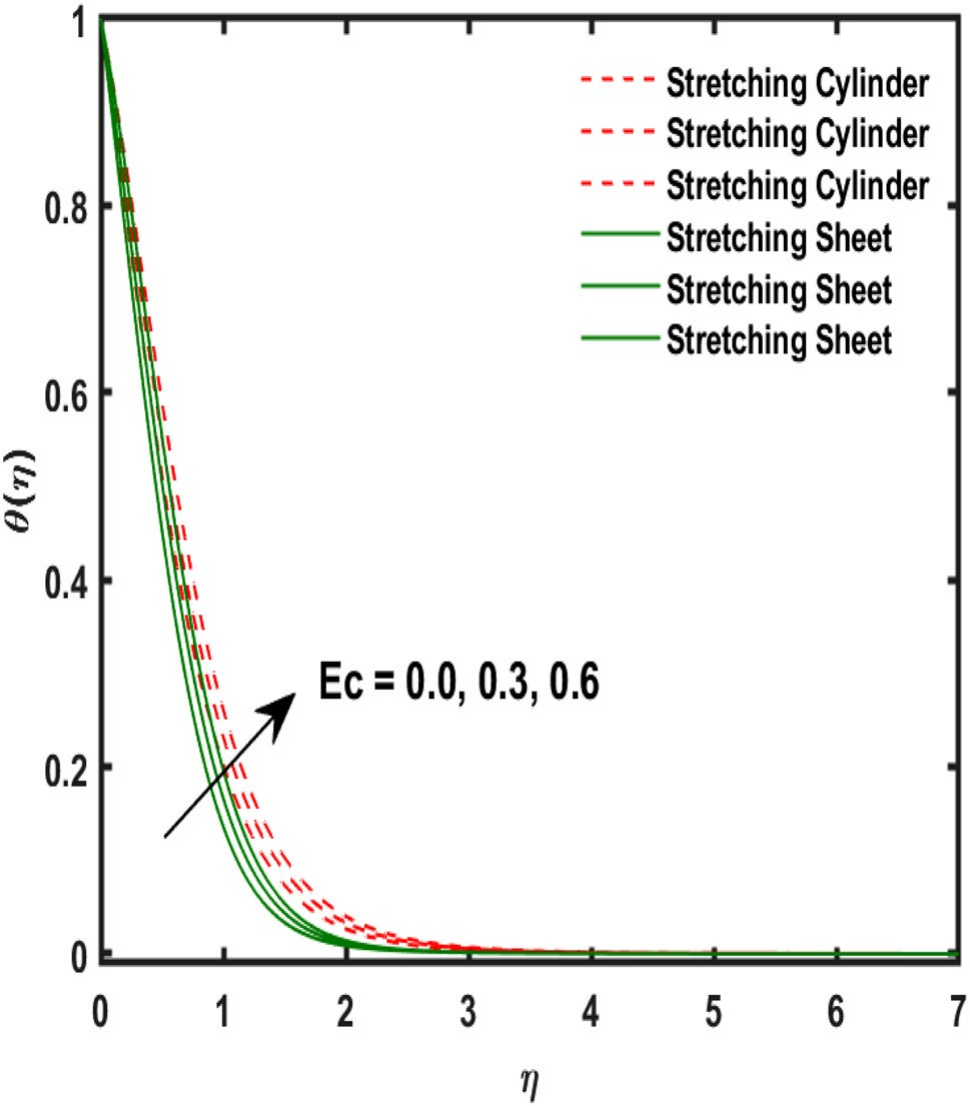
Variation of $Ec$ and $\theta (\eta )$.

**Figure 9:**
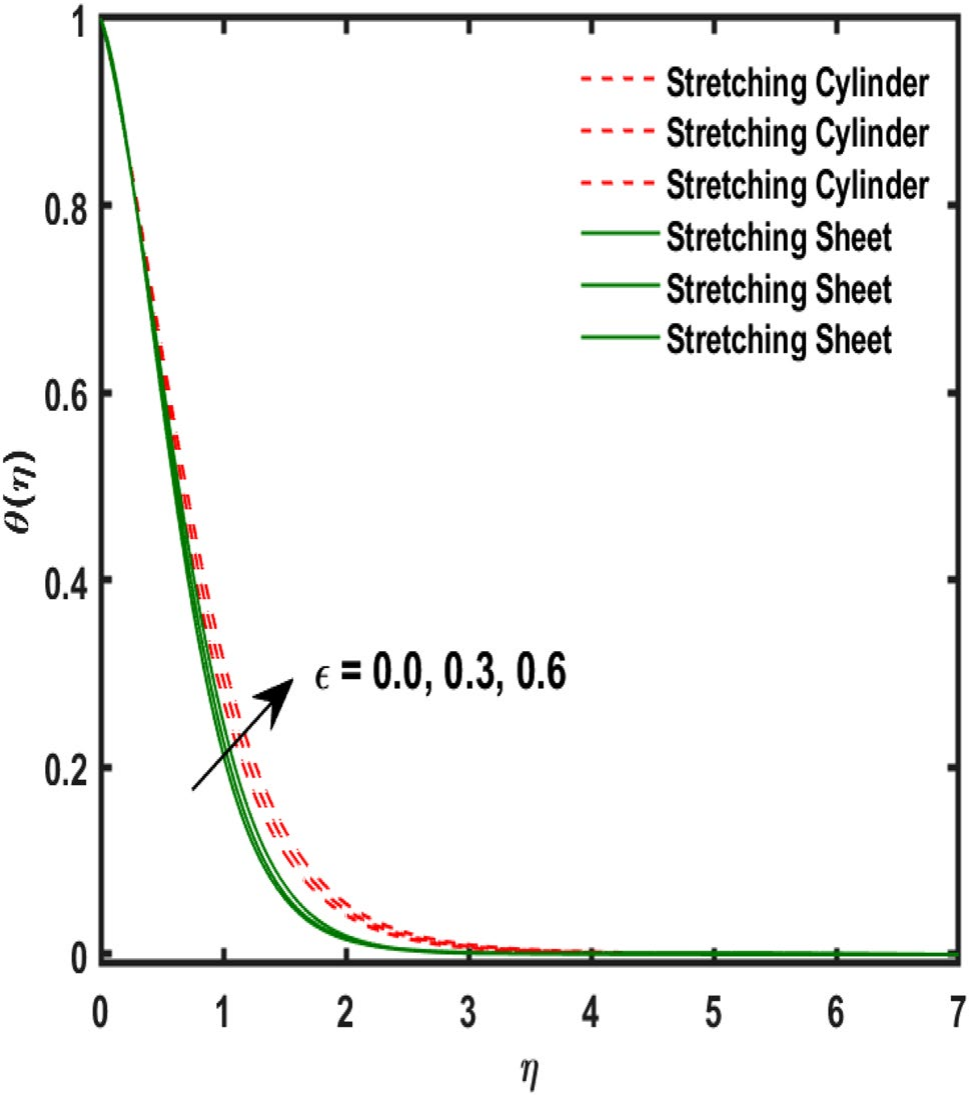
Variation of $\epsilon$ and $\theta (\eta )$.

**Figure 10:**
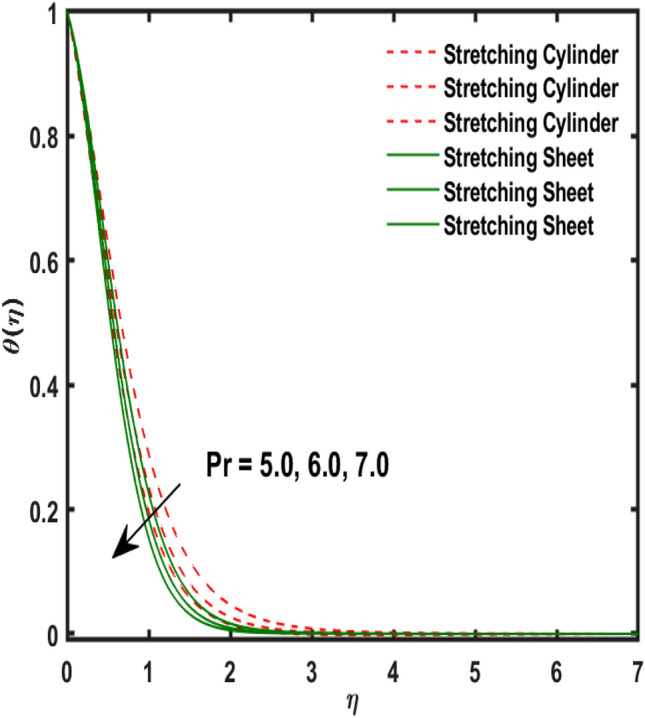
Variation of $Pr$ and $\theta (\eta )$.

**Figure 11:**
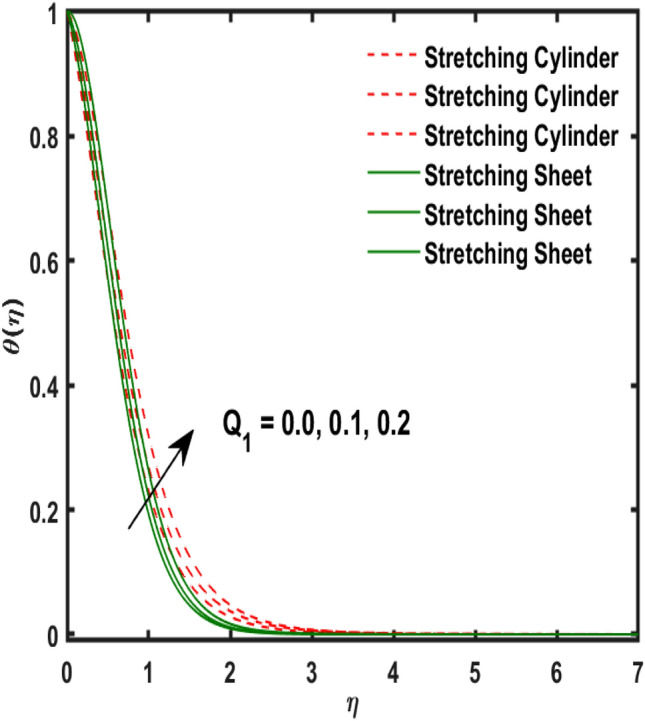
Variation of ${Q}_{1}$ and $\theta (\eta )$.

**Figure 12:**
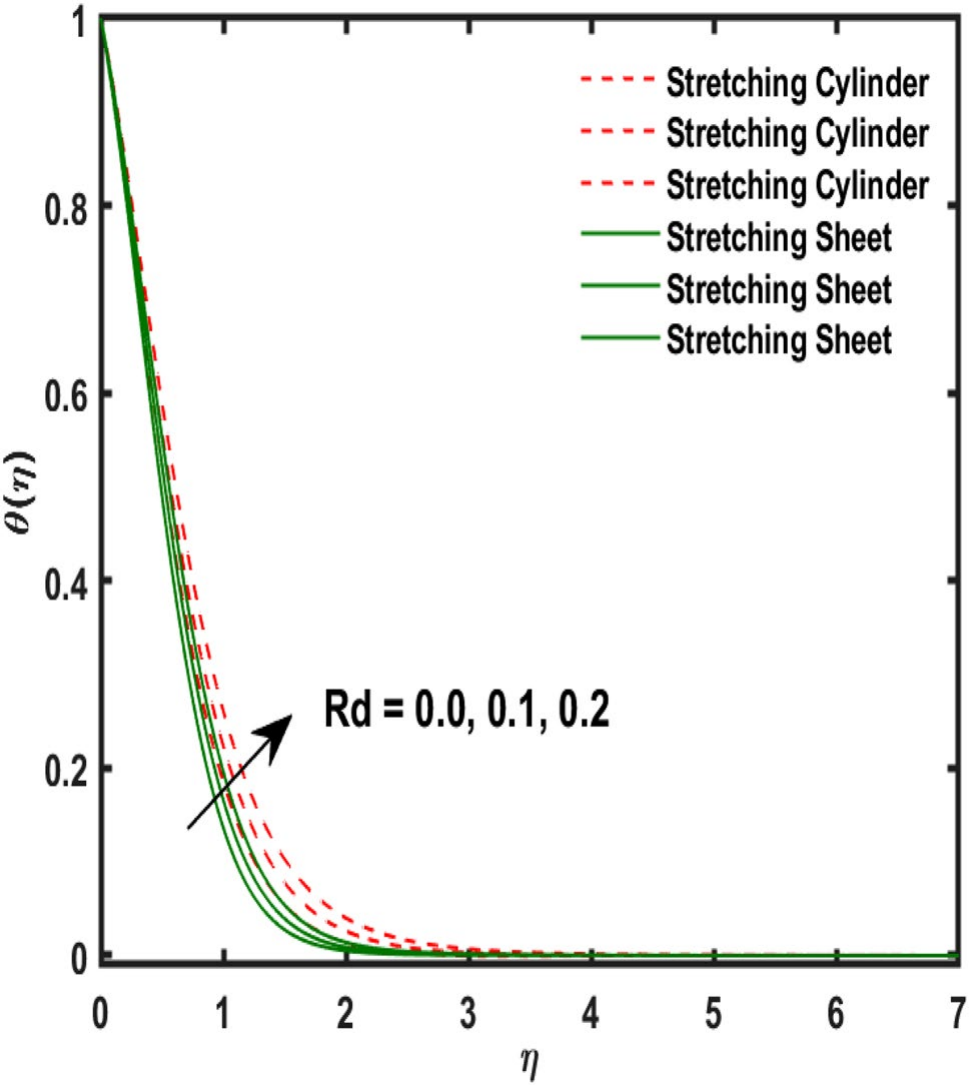
Variation of $Rd$ and $\theta (\eta )$.

The system of dimensionless differential equations are cracked by numerical approach and presented the governing factors are presented through graphs and tabular form. Physical parameter ranges are as $0<\varpi <5$, $0<{\beta }_{1}<2$, $0< {\gamma }_{0}<2$, $0<{\alpha }_{0}<3$, $0<{\alpha }_{1}<3$, $1<{\lambda }_{0}<100$, $0<Ec<2$, $0<\epsilon <2$, $1<Pr<100$, $0<{Q}_{1}<2$ and $0<Rd<3$. These range are taken from the literature which applied in the problem. The impression of involving physical factors namely: ${\beta }_{1}$(Maxwell fluid factor), ${\gamma }_{0}$ (Magnetic field), ${\alpha }_{0}$(Sutterby fluid factor), ${\alpha }_{1}$(Darcy resistant), ${\lambda }_{0}$(Magnetic Prandtl number), $Ec$ (Eckert number), $\epsilon$ (Variable thermal conductivity), $Pr$(Prandtl number), ${Q}_{1}$(Heat generation) and $Rd$(radiation factor) on the temperature, induced magnetic and velocity function which publicised through Figs. 2, 3, 4, 5, 6, 7, 8, 9, 10, 11 and 12.

Figure 2 publicized the variation of ${\alpha }_{1}$((Darcy's resistance) on the velocity. The velocity revealed growing up when the values of ${\alpha }_{1}$(Darcy resistant) boosted up. It relates the frictional pressure drop in porous media to the increase in flow velocity in the media. The variation of ${\alpha }_{0}$ (Sutterby fluid factor) and velocity is publicized in Fig. 3. The velocity revealed deteriorating when the values of ${\alpha }_{0}$ (Sutterby fluid factor) boosted up. Because of the increment in the Sutterby fluid parameter which increment in fluid viscosity ultimately fluid velocity declined because the viscosity of fluid enlarges as well as fluid velocity declined at the surface. Figure 4 exposed the impact of ${\gamma }_{0}$(magnetic field factor) on velocity. The velocity is publicized growing up due to larger values in ${\gamma }_{0}$(magnetic field factor). The velocity profile is boosted by thicker momentum boundary layers, which are caused by higher values of the ${\gamma }_{0}$. Magnetic field is implemented vertically on the fluid flow because Lorentz force is implemented to become a laminar flow. So, the fluid flow become higher velocity due to enlarging values of ${\gamma }_{0}$(magnetic field factor). Figure 5 exposed the impact of ${\beta }_{1}$(Maxwell fluid factor) on velocity. The velocity publicized growing up due to larger values in ${\beta }_{1}$(Maxwell fluid factor). This behaviour is caused by the Maxwell parameter raising the fluid viscosity, which lowers the yield stress, as its values are increased. Variation of ${\gamma }_{0}$(magnetic field factor) and induced magnetic function publicized in Fig. 6. It is noted that induced magnetic curves exposed growing up due to advancing values of ${\gamma }_{0}$(Magnetic field factor). As a result of the magnetic field being applied in the flow norm's direction. The resistive force produced by this magnetic field causes the fluid's velocity to grow up. Variation of ${\lambda }_{0}$(Magnetic Prandtl number) and induced magnetic function publicized in Fig. 7. It is noted that induced magnetic curves revealed deteriorating due to higher values of ${\lambda }_{0}$(Magnetic Prandtl number). The magnetic Prandtl number is the ratio of viscosity to magnetic diffusion. If the viscosity of fluid enhanced which declined the curves of magnetic profile due to enlarging values of magnetic Prandtl number. The physical factor of $Ec$ (Eckert number) and temperature exposed variation in Fig. 8. The temperature curves publicized boosting up due to higher values of $Ec$. The Eckert number enlarged which boosted the viscous heating which improved the temperature of liquid. The physical factor of $\epsilon$ and temperature revealed variation in Fig. 9. The temperature curves publicized boosting up due to higher values of $\epsilon$. The thermal conductivity of liquid boosted which improved the temperature of liquid. Since both kinetic energy and potential collision energy increase with temperature, fluids become more thermally conductive as a result. The thermal conductivity of fluid increases with temperature. The influence of $Pr$(Prandtl number) on the temperature exposed in Fig. 10. Temperature curves exposed declined by addition of $Pr$(Prandtl number) in both cases of stretching cylinder/sheet. Because Prandtl number and thermal conductivity are inversely proportional. As the Prandtl number increased which declined the fluid thermal conductivity ultimately, the temperature of fluid reduced. The influence of ${Q}_{1}$ on the temperature exposed in Fig. 11. Temperature curves exposed increasing by the addition of ${Q}_{1}$(Heat generation) in both cases of stretching cylinder/sheet. Because heat generation built up the temperature high, fluid temperature revealed boost up. Temperature and radiation variation are exposed in Fig. 12. It is realized that temperature curves boost up due to higher values of radiation factor. Because the radiation factor built up the temperature high fluid temperature revealed a boost in both cases of stretching cylinder/sheet. For both heat generation, the temperature field increases for higher values of the radiation parameter. The improvement in temperature distribution is caused by higher values of Rd.

Table 1 incorporated the influence of physical factors namely: $Rd$(radiation factor), $Pr$(Prandtl number), ${Q}_{1}$(Heat generation), $Ec$(Eckert parameter), $\epsilon$(small parameter), ${\gamma }_{0}$(Magnetic field factor), ${\lambda }_{0}$(Magnetic Prandtl number), ${\alpha }_{0}$(Sutterby fluid factor), ${\alpha }_{1}$(Darcy resistant) and ${\beta }_{1}$(Maxwell fluid factor) on the friction factor and heat transfer factor. Friction feature degenerated while heat transfer factor advanced due to increment of radiation factor in both cases of stretchable sheet/cylinder. Because the radiation factor produced heat increment due to higher values of radiation factor which improved the heat transfer phenomena enhance. Friction feature is fixed while heat transfer factor degenerated due to an increment of Prandtl number in both cases of stretchable sheet/cylinder. Physically, thermal conductivity of liquid degenerated due to enlarging values of $Pr$, ultimately heat transfer phenomena revealing decline at surface. Friction feature is enlarged while heat transfer factor advanced up due to an increment of heat generation factor in both cases of stretchable sheet/cylinder. Because the heat generation factor produced heat increment due to higher values of heat generation factor which improved the heat transfer phenomena enhance. The friction feature and heat transfer factor advanced due to the addition of heat Eckert number in both cases of stretchable sheet/ cylinder. Because, Eckert number produced heat increment which developed the heat transfer phenomena augment. Physically, the kinematic energy of fluid particles boosted due to enlarging the values of Eckert number ultimately, heat transfer factor enlarged. Due to random motion of fluid particles for enlarging values of Eckert number ultimately, the friction feature increased. The heat transfer factor heightened while friction feature advanced up due to increment of $\epsilon$ in both cases of stretchable sheet/cylinder. Temperature depends on the thermal conductivity of the material and has directly proportional relation. As thermal conductivity boosted due to enlarging values of thermal conductivity parameter ultimately, temperature of fluid revealed enhancement. Heat transfer factor and friction at surface factor boosted up for increment of ${\gamma }_{0}$. The ${\gamma }_{0}$(Magnetic field factor) is larger which better-quality of heat transfer at surface and also offered the results of friction factor boosting up in both cases of stretching sheet/cylinder. The ${\lambda }_{0}$(Magnetic Prandtl number) increased which provided better-quality of heat transfer at surface. It also offered the results of friction factor boosting up in case of stretching cylinder but opposite behaviour have been noted for both Heat transfer factor and friction at surface factor degenerated due to larger values of ${\lambda }_{0}$ in case of stretching sheet. Increment of ${\alpha }_{0}$(Sutterby fluid factor) which improved friction and heat transfer factors for both stretching cylinder/sheet cases. Because, the increment in Sutterby fluid parameter which increment in fluid viscosity ultimately fluid velocity declined because viscosity of fluid enlarges as well as friction boosted up at surface. The ${\alpha }_{1}$(Darcy resistant) larger which better-quality of heat transfer at surface. It is also offered the results of friction factor deteriorating in case of stretching cylinder but opposite behaviour have been noted for both Heat transfer factor and friction at surface due to larger values of ${\alpha }_{1}$(Darcy resistant) in case of stretching sheet. It relates the frictional pressure drop in porous media to the increase in flow velocity in the media ultimately, friction dropped at surface of cylinder and opposite behaviour have been noted for both Heat transfer factor and friction at surface due to larger values of ${\alpha }_{1}$(Darcy resistant) in case of stretching sheet. The ${\beta }_{1}$(Maxwell fluid factor) enlarged which better-quality of heat transfer at surface and also offered the results of friction factor boosting up in both cases of stretching sheet/cylinder. The friction factor is boosted up due to thickness of momentum improved which this behaviour is caused by the Maxwell parameter raising the fluid viscosity, which lowers the yield stress, as its values are increased. The comparative analysis of the present model is done in Tables 2, 3. It is noted that the present work has been found to be good agreement with Rangi and Ahmad^45^, Qasim et al.^47^ and Suleman et al.^46^. We compared the results of $-\theta {\prime}(0)$ (Rangi and Ahmad^45^) for different values of $\varpi$ and rest of the values are zero and $Pr=1.0$ in Table 2. We compared the results of $-\theta {\prime}(0)$ (Qasim et al.^47^ and Suleman et al.^46^) for different values of $Pr$ and rest of the values are zero with $\varpi =1.0$ and $\varpi=0.0$ in Table 3.

**Table 1:** Variation of Skin friction and Heat transfer factor with involving physical factors.

										Stretching cylinder $\varpi \ne 0$		Stretching sheet $\varpi =0$	
$Rd$	$Pr$	${Q}_{1}$	$Ec$	$\epsilon$	${\gamma }_{0}$	${\lambda }_{0}$	${\alpha }_{0}$	${\alpha }_{1}$	${\beta }_{1}$	$F{\prime}{\prime}(0)$	${{N}^{n}}_{u}$	$F{\prime}{\prime}(0)$	${{N}^{n}}_{u}$
0.0	5.0	0.3	0.4	0.2	0.3	0.3	0.5	0.4	0.2	0.06445934	0.8639177	0.7675782	1.75589
0.2										0.06445889	0.9818927	0.7675741	1.955666
0.4										0.06445873	1.0877230	0.7675653	2.128006
0.6										0.06445868	1.1853250	0.7675450	2.281536
0.2	5.0									0.06445889	1.0256660	0.7675741	2.079936
	6.0									0.06445889	0.9818927	0.7675741	1.955666
	7.0									0.06445889	0.9389360	0.7675741	1.846399
	8.0									0.06445889	0.8987807	0.7675741	1.749690
	5.0	0.0								0.06445836	0.3217396	0.7675732	1.347418
		0.3								0.06445889	0.9818927	0.7675741	1.955666
		0.6								0.06445915	1.4675330	0.7675750	2.397297
		0.9								0.06445935	1.8630540	0.7675763	2.758506
		0.3	0.0							0.05762800	0.7646822	0.7675725	0.8113493
			0.2							0.06445887	0.8747556	0.7675736	1.3779190
			0.4							0.06445889	0.9818927	0.7675741	1.9556660
			0.6							0.06445935	1.0935530	0.7675748	2.5453090
			0.4	0.0						0.06445835	0.9818927	0.7675736	1.955666
				0.2						0.06445889	1.0645260	0.7675741	2.185040
				0.4						0.06445895	1.1373920	0.7675748	2.396661
				0.6						0.06445935	1.2018740	0.7675754	2.593302
				0.2	0.1					0.01290980	0.3747460	0.9933079	1.816793
					0.3					0.06445880	0.9818927	0.7675741	1.955666
					0.6					0.11931900	1.5041390	3.078581	2.603245
					0.9					0.17871400	1.5944260	6.697452	7.078156
					0.3	0.1				0.01744480	0.4364010	1.709155	2.6379411
						0.3				0.06445880	0.9818927	0.7675741	1.955666
						0.5				0.15991500	1.4196320	0.1501416	1.587469
						0.7				0.25615100	2.4144590	0.0346374	0.755850
						0.3	0.0			0.00901080	0.3949510	-1.224454	1.720974
							0.5			0.06445889	0.9818927	0.7675741	1.955666
							1.0			1.58577400	1.6806630	1.420893	2.082659
							1.5			1.98997270	1.9847200	1.907129	2.111337
							0.5	0.0		1.17169100	1.4200270	0.7044543	0.940935
								0.2		1.06559700	1.4170970	0.7214939	1.023533
								0.4		0.06445889	0.9818927	0.7675741	1.955666
								0.6		0.01582570	0.4218530	1.5578700	2.024054
								0.4	0.0	0.01926460	0.4452780	0.090084	1.533875
									0.2	0.06445889	0.9818927	0.4275741	1.955666
									0.4	1.72461700	1.3320069	1.721659	2.161398
									0.6	1.78977200	2.5185720	1.918714	3.3804561

**Table 2:** Validation results of Heat transfer factor with involving physical factors and rest of parameters are $Rd={Q}_{1}=Ec={\gamma }_{0}={\lambda }_{0}={\alpha }_{0}={\alpha }_{1}={\beta }_{1}=0$ and $Pr=1.0$.

$\varpi$	Present results ($-\theta {\prime}(0)$)		Rangi and Ahmad^45^ ($-\theta {\prime}(0)$)	
	$\epsilon =0.0$	$\epsilon =0.2$	$\epsilon =0.0$	$\epsilon =0.2$
0.00	–0.985286	–0.862122	–0.9846372	–0.861768
0.25	–1.079447	–0.949659	–1.0787951	–0.948723
0.50	–1.173899	–1.037605	–1.1728978	–1.036756
0.75	–1.267214	–1.124397	–1.2667873	–1.123578
1.00	–1.359308	–1.209949	–1.3585371	–1.209785

**Table 3:** Validation results of Heat transfer factor with involving physical factors and rest of parameters are $Rd={Q}_{1}=Ec={\gamma }_{0}={\lambda }_{0}={\alpha }_{0}={\alpha }_{1}={\beta }_{1}=0$.

$Pr$	$\varpi$	Qasim et al.^47^	Suleman et al.^46^	Present results
0.720	0.00	1.2366400	1.2366510	1.225743
1.000		1.0000000	1.0000000	1.000000
6.700		0.3333000	0.3333100	0.333304
10.00		0.2687600	0.2687700	0.267962
0.720	1.00	0.8701800	0.8701900	0.868951
1.000		0.7440600	0.7440700	0.737862
6.700		0.2966100	0.2966200	0.287683
10.00		0.2421700	0.2421800	0.239752

## Conclusion

Steady incompressible sutterby Maxwell fluid flow at stretching cylindrical surface is taken into account. Radiation and joule heating influence are studied under the temperature dependent properties of liquid. The induced magnetic field is considered in present analysis. The main key finding results are presented below:

- The velocity profile is boosted by thicker momentum boundary layers, which are caused by higher values of the ${\gamma }_{0}$.$-$
- The friction factor is boosted due to improving the Maxwell fluid parameter. This behaviour is caused by the Maxwell fluid parameter raising the fluid viscosity, which lowers the yield stress, as its values are increased.
- Increment of ${\alpha }_{0}$(Sutterby fluid factor) which improved friction and heat transfer factors for both stretching cylinder/sheet cases. Because, the increment in Sutterby fluid parameter which increment in fluid viscosity ultimately fluid velocity declined because viscosity of fluid enlarges as well as friction boosted up at surface.
- The velocity revealed deteriorating when the values of ${\alpha }_{0}$(Sutterby fluid factor) boosting up. Because, the increments in Sutterby fluid parameter which increment in fluid viscosity ultimately fluid velocity declined because viscosity of fluid enlarges as well as fluid velocity declined at surface.
- $$-$$

## Data Availability

The datasets generated during the current study are not publicly available but are available from the corresponding author on reasonable request.

## List of symbols

$$\varpi$$: Curvature parameter (1)
${\alpha }_{0}$: Sutterby fluid factor (1)
$$Ec$$: Eckert number (1)
$${Q}_{1}$$: Heat generation (1)
$$\theta \left(\eta \right)$$: Temperature profile (1)
$r$: Radial direction (coordinate) (m)
$x$: Axial direction (coordinate) (m)
$T$: Temperature component (kelvin)
$${U}_{w}$$: Wall velocity (m/s)
$${\eta }_{0}$$: Magnetic diffusivity (m^2^/s)
$${\mu }_{e}$$: Magnetic permeability (N⋅A^−2^)
$$\rho$$: Density (kg/$${m}^{3}$$)
${Q}_{0}$: Coefficient of Heat generation
${\gamma }_{0}$: Magnetic field factor (1)
${\lambda }_{0}$: Magnetic Prandtl number (1)
$$Pr$$: Prandtl number (1)
$\epsilon$: Variable thermal conductivity (1)
$$\eta$$: Dimensionless variable (1)
${\psi }_{1}$: Stream function of induced magnetic field (kg/ms)
$${v}_{1}$$: Velocity component (m/s)
${\beta }_{1}$: Maxwell fluid factor (1)
$${\alpha }_{1}$$: Darcy resistant (1)
$$Rd$$: Radiation factor (1)
$$F\left(\eta \right)$$: Velocity profile (1)
$G\left(\eta \right)$: Induced magnetic profile (1)
$\psi$: Stream function of velocity
$${u}_{1}$$: Velocity component (m/s)
${H}_{2}$: Induced magnetic component (m/s)
${T}_{w}$: Wall temperature (kelvin)
$${\upsilon }_{0}$$: Kinematic viscosity ($${m}^{2}/s$$)
$$Re$$: Reynolds number (1)
${a}_{0}$: Consistency index (Pa.s^n^)
$$\mu$$: Dynamic viscosity ($$kg/ms$$)
${H}_{1}$: Induced magnetic component (m/s)
${T}_{\infty }$: Ambient temperature (kelvin)
${H}_{e}$: Free stream magnetic field (kg/ms)
$$\tau$$: Relaxation time
$$m$$: Flow index (1)
$${c}_{p}$$: Heat capacity $$({m}^{2}/{s}^{-2}{k}^{-1})$$
$a$: Length of stretching sheet (1)

## Abbreviations

MHD: Magnetic hydrodynamic
ODE’s: Ordinary differential equations
R-K-F: Runge–Kutta-Fehlberg
PDE’s: Partial differential equations
